# Fungal resilience and host–pathogen interactions: Future perspectives and opportunities

**DOI:** 10.1111/pim.12946

**Published:** 2022-08-27

**Authors:** Alistair J. P. Brown

**Affiliations:** ^1^ Medical Research Council Centre for Medical Mycology at the University of Exeter Exeter UK

**Keywords:** antifungal drug resistance, antifungal immunity, fungal pathogenicity, fungal resilience, host–pathogen interactions, metabolic adaptation, stress resistance

## Abstract

We are constantly exposed to the threat of fungal infection. The outcome—clearance, commensalism or infection—depends largely on the ability of our innate immune defences to clear infecting fungal cells versus the success of the fungus in mounting compensatory adaptive responses. As each seeks to gain advantage during these skirmishes, the interactions between host and fungal pathogen are complex and dynamic. Nevertheless, simply compromising the physiological robustness of fungal pathogens reduces their ability to evade antifungal immunity, their virulence, and their tolerance against antifungal therapy. In this article I argue that this physiological robustness is based on a ‘Resilience Network’ which mechanistically links and controls fungal growth, metabolism, stress resistance and drug tolerance. The elasticity of this network probably underlies the phenotypic variability of fungal isolates and the heterogeneity of individual cells within clonal populations. Consequently, I suggest that the definition of the fungal Resilience Network represents an important goal for the future which offers the clear potential to reveal drug targets that compromise drug tolerance and synergise with current antifungal therapies.

## 1 INTRODUCTION

The interactions between fungal pathogens and their hosts are both complex and dynamic. The complexity arises largely from the evolutionary diversity of fungal pathogens, the numerous niches that these pathogens can occupy within us and from the multifarious types of host cell that contribute to antifungal immunity. The dynamism of host–fungus interactions is enhanced by host microenvironments that are in constant state of flux. These microenvironments change continuously as the fungus adapts and grows within them, as immune cells infiltrate to promote fungal killing and clearance and as the fungus then responds to these host defences. In this article, I contend that it is vital that we develop a deep understanding of host–fungus interactions, including their complexity and dynamism, because these interactions ultimately determine the outcome for the fungus and, consequently, the colonized host. Furthermore, a thorough understanding of these interactions is likely to reveal powerful therapeutic strategies that will favour positive outcomes for patients.

Bernhard Hube, a renowned expert in the field, has drawn an ineluctable conclusion about host–fungus interactions: he says ‘*It's complicated!*’. Paul Nurse also states that life is complex. He suggests that we first need to *describe* the complexity of living systems and then *understand* this complexity.^1^ Nurse goes on to argue that we should seek simplicity from this complexity and that abstractions offered by systems‐based approaches may offer an approach towards achieving this.^1^

Bearing this in mind, one major challenge seems clear: we need to understand ‘fungal resilience’. This view is based on the hypothesis that the expression of critical phenotypes that drive fungal pathogenicity and that compromise antifungal therapy—virulence factors, immune evasion strategies, the emergence of antifungal drug resistance and tolerance—are strongly influenced by the physiological status of the fungal cell. This ‘physiological status’ underlies the resilience of a fungal cell to environmental challenges such as changes in nutrient availability, the imposition of stresses and exposure to antifungal drugs.

**FIGURE 1:**
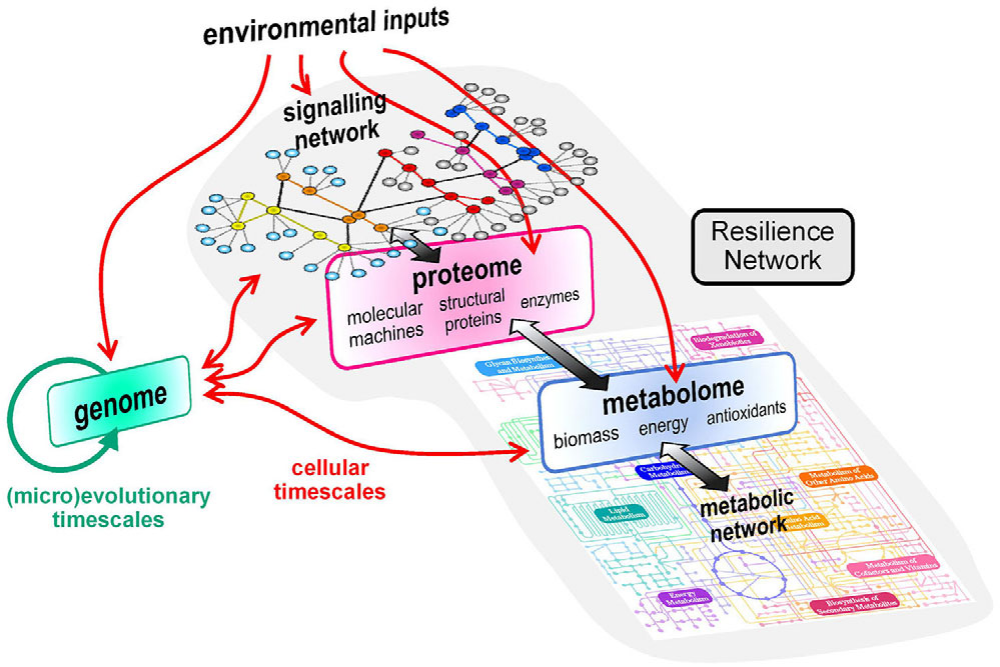
The concept of the ‘Resilience Network’. The fungal Resilience Network represents those features that promote the physiological robustness of the fungal cell as it responds to environmental inputs and genetic variation. In cellular timescales, environmental inputs such as stresses and changes in nutrient availability are perceived by the cell and these signals transduced via a signalling network. This signalling network ultimately links all signal transduction pathways (in the same way that all metabolic pathways are ultimately linked via the metabolic network) and this imparts elasticity and robustness through compensatory changes within the Resilience Network. The signalling network triggers changes in gene expression as well as modulating the activities of proteins within the proteome. This in turn mediates changes in the metabolic network and metabolome. Changes in the metabolome also feedback to the proteome, the signalling network and the genome. In (micro)evolutionary timescales, some environmental inputs cause mutations which, when combined with replication errors, lead to genetic variation. Some mutations will influence the functionality of the signalling network and proteome and thereby the functionality of the Resilience Network

By analogy with the metabolic network, the signalling pathways that regulate the growth, metabolism and drug and stress responses of fungal pathogens are all linked within a large ‘Resilience Network’ (Figure 1). Like the metabolic network, this Resilience Network contains hubs, such as the target of rapamycin (TOR), protein kinase A (PKA), protein kinase C (PKC), AMP kinase, cyclin‐dependent kinase, Hog1 and Hsp90, each of which exerts a strong influence over the growth status and physiological robustness of the fungal cell. Fungal pathogens differ with respect to their resistance to environmental challenges.^2^ Nevertheless, there is strong evidence to indicate that compromising this Resilience Network attenuates fungal virulence, affects immune evasion strategies and diminishes antifungal drug resistance and tolerance.^3^, ^4^, ^5^, ^6^, ^7^, ^8^, ^9^, ^10^, ^11^, ^12^ Therefore, in this perspective, I first set the scene by discussing the diversity of fungal pathogens and then describe the concept of the Resilience Network and the likely conservation of key components of this Network across fungal pathogens. The challenge to define the structure and dynamics of this network in fungal pathogens is then addressed, together with speculation about possible future advances that may empower this.

## 2 UNDERLYING SIMILARITIES IN IMMUNE EVASION BETWEEN EVOLUTIONARILY DIVERSE FUNGAL PATHOGENS

Fungal pathogens display great evolutionary diversity and, thanks to their different lifestyles, are on different evolutionary trajectories. Pathogenicity traits that promote infections in animals and humans have emerged independently in different phylogenetic branches of the fungal tree of life. For example, *Cryptococcus neoformans* and *Rhizopus delemar* are members of the Basidiomycota and Mucoromycota, respectively and *Pneumocystis jirovecii*, *Histoplasma capsulatum*, *Talaromyces marneffei*, *Sporothrix schenckii*, *Aspergillus fumigatus* and *Candida albicans* belong to different families within the Ascomycota. Many of these species are saprotrophs, existing mainly in the environment and yet they have independently honed phenotypes that promote infection in humans (e.g. *T. marneffei*, *S. schenckii*, *A. fumigatus*). In contrast, other fungal pathogens appear to have co‐evolved with their host to become commensals (e.g. *C. albicans*) or even to the extent that they have become obligately dependent on their human host for their survival (e.g. *P. jirovecii*). Therefore, fungal pathogens are evolving in different niches, they display a high degree of genetic and phenotypic diversity and they differ significantly with respect to the degree to which they have co‐evolved with their human host. This complexity is further enhanced by the genetic and phenotypic variability displayed by individual isolates and even by the phenotypic heterogeneity of clonal populations of fungal pathogens.^13^, ^14^, ^15^

This all suggests that fungal pathogens of humans have been subjected to quite different evolutionary pressures. Nevertheless, a core set of fundamental properties is required to colonize and infect humans and this includes the ability to avoid or evade antifungal immunity. How might environmental saprotrophs, which do not normally encounter humans, have evolved the ability to evade immunity? Some amoebae are able to feed on fungi,^16^, ^17^ which led to the attractive idea that the evolution of resistance to amoebic predation in the environment might have accidentally promoted phenotypes in *C. neoformans* that enhance its resistance to phagocytic killing in humans,^18^ and this idea has been extended to other saprobic fungal pathogens.^19^, ^20^

Phagocytic killing can be avoided in a number of ways, for example, by activating stress responses that counter phagocytic killing mechanisms, by preventing phagolysosomal maturation and hence the activation of these killing mechanisms or by avoiding phagocytic uptake in the first place. These strategies resonate with those employed by many fungal pathogens in humans. Therefore, despite dramatic differences between niches in the environment and in humans, some common selective pressures are likely to have fortuitously promoted the pathogenicity of some saprobic fungi.

Predictably, given their evolutionary diversity, fungal pathogens have developed a diverse range of evasion strategies. For example, *Blastomyces dermatitis* generally evades amoebic uptake, whereas *H. capsulatum* is quickly engulfed by amoebae and then thrives intracellularly, while *A. fumigatus* is phagocytosed but subsequently kills the predatorial amoeba.^20^, ^21^ Casadevall argues that while some phenotypes that protect fungi against amoebic predation may promote virulence in humans, the reverse might not hold true.^22^ For example, he suggests that fungal factors that modulate immune function in humans are unlikely to be relevant in amoebae given their lack of an immune system.^22^ Interestingly, *C. albicans* is killed by amoebae,^23^ indicating that the immune evasion strategies that this fungus has developed during its co‐evolution with the host are unlikely to protect it from amoebic predation in the environment.

### 2.1 Concealment

*C. albicans* has evolved an impressive armoury of immune evasion strategies some of which have parallels in other evolutionarily divergent fungal pathogens.^24^, ^25^, ^26^ The first of these strategies involves concealment—mechanisms that limit fungal recognition by innate immune cells.

Much emphasis has been placed on the concealment of the proinflammatory pathogen‐associated molecular pattern (PAMP), β‐(1,3)‐glucan. The polymer, β‐(1,3)‐glucan, is an essential component of the cell walls of many fungi.^27^ In *C. albicans*, most β‐(1,3)‐glucan is present in the inner cell wall, buried beneath the outer layer of mannan fibrils,^28^ but some β‐(1,3)‐glucan becomes exposed at septal junctions between mother‐daughter cells, at bud scars and at punctate foci on the cell surface.^29^ Also, β‐(1,3)‐glucan on the lateral cell wall can become exposed in vivo through stripping of the mannan outer layer by neutrophil attack.^30^ To promote concealment, *C. albicans* secretes an exoglucanase (Xog1) and an endoglucanase (Eng1) that shave exposed β‐(1,3)‐glucan from the cell surface.^31^, ^32^ Interestingly, *C. albicans* induces β‐(1,3)‐glucan masking in response to host signals that potentially forewarn of impending attack by innate immune cells: lactate, hypoxia, iron limitation and ambient pH.^11^, ^13^, ^31^, ^33^, ^34^, ^35^ β‐(1,3)‐Glucan shaving from the cell surface correlates with reduced recognition by innate immune cells and attenuated cytokine responses.^11^, ^13^, ^34^ This does, however, depend on the type of innate immune cell. While mannan acts to dampen β‐(1,3)‐glucan‐mediated antifungal responses in macrophages, mannan induces antifungal responses in monocytes.^36^ Nevertheless, these observations have led to the idea that, over evolutionary time, *C. albicans* has developed a ‘memory’ of stresses that are likely to follow specific host signals and, consequently, has evolved the ability to anticipate immune attack and activate pre‐emptive protective responses.^35^

PAMP concealment is also employed by other fungal pathogens. For example, *H. capsulatum* masks β‐(1,3)‐glucan beneath a layer of α‐(1,3)‐glucan and expresses an Eng1 endoglucanase that trims exposed β‐(1,3)‐glucan and helps this pathogen ‘fly under the radar’ of immune surveillance.^37^, ^38^, ^39^, ^40^ Meanwhile, *C. neoformans* synthesizes an extracellular capsule, which not only conceals cell wall PAMPs but also induces the anti‐inflammatory cytokine IL‐10, thereby dampening antifungal responses.^41^, ^42^, ^43^ Likewise, *A. fumigatus* conceals the β‐(1,3)‐glucan in its spore cell wall underneath a layer of the RodA hydrophobin.^44^ Clearly, PAMP concealment is a common strategy employed by evolutionary divergent fungal pathogens.

### 2.2 Manipulation

The manipulation of antifungal immune defences provides a complementary strategy to concealment.^25^ For example, *C. albicans* secretes aspartic proteases that inactivate complement proteins.^45^ *C. albicans*, *A. fumigatus* and *C. neoformans* secrete proteins that interfere with the functionality of the complement system or complement receptors, thereby reducing phagocytic uptake.^46^, ^47^, ^48^, ^49^, ^50^, ^51^ Furthermore, *C. albicans*, *Paracoccidioides brasiliensis*, *C. neoformans* and *A. fumigatus* all express proteins that bind plasminogen to interfere with complement activation.^25^, ^52^, ^53^, ^54^

Fungal pathogens can manipulate host immunity even after phagocytic uptake, by inhibiting phagolysosomal maturation. For example, *C. neoformans*, *Candida glabrata* and *H. capsulatum* are able to persist within innate immune cells by blocking phagosomal maturation or acidification.^55^, ^56^, ^57^, ^58^, ^59^ Although the outcome is similar, these pathogens exploit different strategies to achieve persistence inside macrophages. *H. capsulatum* and *C. glabrata* persist by blocking phagolysosomal acidification, whereas *C. neoformans* survives in acidic phagolysosomes.^55^, ^58^, ^60^

*C. albicans* was thought to drive metabolic alkalinization to help maintain a neutral pH within the phagosome and escape phagocytic killing.^61^, ^62^, ^63^ More recent data suggest that alternative mechanisms may drive alkalinization,^64^, ^65^, ^66^ including yeast‐hypha morphogenesis which can lead to rupture of the phagosome and death of the macrophage.^67^, ^68^, ^69^ Macrophages attempt to overcome hyphal development and fungal escape by physically folding hyphae.^70^ However, *C. albicans* can trigger pyroptosis, inflammasome activation and death of the macrophage and can also promote immune cell death through competition for glucose.^71^, ^72^, ^73^, ^74^, ^75^

### 2.3 Resistance

In addition to concealment and manipulation, fungal pathogens exploit resistance mechanisms to counteract antifungal killing mechanisms.^24^, ^25^, ^26^ Innate immune cells exploit a battery of fungicidal mechanisms that include toxic chemicals, cationic fluxes, enzymes and antimicrobial peptides.^76^ In particular, the respiratory burst generates high concentrations of reactive oxygen species (ROS) that would be lethal for many fungal species.^2^ However, fungal pathogens such as *C. albicans*, *C. glabrata*, *C. neoformans* and *H. capsulatum* display robust responses to oxidative stress that provide some degree of protection against the respiratory burst.^2^, ^7^, ^77^, ^78^, ^79^ Indeed, *C. albicans* and *H. capsulatum* even express superoxide dismutases on their cell surface to detoxify extracellular ROS.^79^, ^80^, ^81^ In addition, fungal pathogens such as *C. albicans* have evolved mechanisms that protect against cationic fluxes, reactive nitrogen species and antimicrobial peptides.^7^, ^82^, ^83^, ^84^

To summarize, despite their evolutionary diversity and their different lifestyles, many fungal pathogens have developed similar approaches that help them to evade antifungal immunity. A second important point, which is pertinent to this review, is that many mechanisms that promote concealment, manipulation and resistance are environmentally, and in some cases, developmentally contingent. For example, the glucanases that conceal the proinflammatory PAMP β‐(1,3)‐glucan are regulated in response to specific environmental inputs and yeast‐hypha morphogenesis in *C. albicans* and, in *H. capsulatum*, are expressed by the pathogenic yeast form.^11^, ^13^, ^39^ The robust stress responses that contribute to resistance against phagocytic killing are strongly influenced by carbon source in *C. albicans*.^85^, ^86^, ^87^ These and many other observations indicate that the signalling mechanisms that regulate many immune evasion strategies are integral to the Resilience Network that governs responses to environmental change^11^, ^13^, ^86^, ^87^, ^88^, ^89^ (Figure 1).

## 3 RELATED FUNGAL VIRULENCE FACTORS ACTIVATED DURING ENVIRONMENTAL ADAPTATION

The same holds true for the ‘Virulence Factors’ that promote fungal infections in humans. The signalling pathways that control the expression of these factors are interconnected with those that drive the adaptive responses to environmental change.^90^ This view is supported by numerous examples in many different fungal pathogens.

Cellular morphogenesis is a major virulence factor. The regulation of morphogenesis and virulence by environmental inputs is particularly obvious for dimorphic fungal pathogens such as *H. capsulatum*.^91^, ^92^ An increase in ambient temperature to 37°C triggers the morphological transition from the environmental filamentous form to the pathogenic yeast form and also the expression of the calcium binding protein, Cpb1 and the cell surface protein, Yps3,^93^ both of which are required for virulence. The formation of large polyploid Titan cells by *C. neoformans*, which display altered PAMP exposure, physical evasion of phagocytosis and elevated drug resistance, is triggered by a variety of environmental stimuli, including nutrient starvation, hypoxia, bacterial peptidoglycan and quorum sensing molecules.^94^, ^95^, ^96^ Significantly Titan formation is regulated by protein kinase A (PKA) signalling.^94^ PKA signalling also drives yeast‐hypha morphogenesis in *C. albicans* in response to environmental signals that include temperature, serum, neutral pH, nutrient starvation, hypoxia, CO_2_, bacterial peptidoglycan and release from quorum sensing.^97^, ^98^, ^99^, ^100^, ^101^, ^102^ Significantly, key regulators of yeast‐hypha morphogenesis in *C. albicans* also control metabolism (e.g. Efg1, Nrg1, Tup1).^103^, ^104^ For example, Efg1 regulates genes involved in glycolysis and oxidative phosphorylation,^103^ whereas Nrg1 and Tup1 repress genes required for the uptake of some carbon sources and the glyoxylate cycle.^104^ Furthermore, hyphal development is controlled by intracellular signals such as the inhibition of cell cycle progression, as well as by extracellular stimuli,^105^ and core stress and growth regulators modulate hyphal development (e.g. Tor1, Hog1).^3^, ^106^

The ability to switch between different phenotypic forms—white, opaque, grey—also promotes the fitness of *C. albicans* in different host niches.^102^, ^107^, ^108^, ^109^, ^110^ Phenotypic switching is regulated by a complex transcriptional circuit in which Efg1 and Wor1 play central roles in controlling the different morphologies, mating capacities, cell walls and metabolism of white, grey and opaque cells.^111^, ^112^ Phenotypic switching is induced by environmental stimuli such as temperature, CO_2_, hypoxia and environmental stresses.^113^, ^114^, ^115^

The control of morphogenesis and phenotypic switching highlights the interconnectedness of developmental, environmental and growth regulation in *C. albicans* and this concept extends to many other fungal virulence factors. The fungal cell wall is viewed as a virulence factor because it is the first point of physical contact between fungus and host and because it provides a robust shield against host‐imposed stresses.^27^ The cell wall is a flexible and elastic structure that, in *C. albicans*, responds to genetic and environmental challenges via the cell wall remodelling, calcium and Hog1 signalling pathways.^86^, ^116^ It is also sensitive to nutrient and micronutrient availability.^11^, ^86^, ^117^, ^118^ In *C. albicans*, the secretion of aspartic proteases (SAPs) also depends upon nutrient availability.^119^ Many adhesins (e.g. Als3, Hwp1), the Als3 invasin and the toxin candidalysin (encoded by *ECE1*) are expressed during hyphal development.^120^, ^121^, ^122^, ^123^, ^124^ Therefore, the regulation of these virulence factors is intimately associated with the signals that trigger yeast‐hypha morphogenesis (above).

Analogous observations have been made about the regulation of virulence factors in other fungal pathogens. For example, capsule formation in *C. neoformans* is regulated by iron limitation, nutrient depletion and serum, in part by PKA and Rim101 signalling.^125^, ^126^, ^127^ pH signalling via the transcription factor Rim101 in *C. albicans* and via its orthologue PacC in *A. fumigatus*, is critical for the virulence of these fungi.^128^, ^129^, ^130^, ^131^ Also, the expression of toxins in other fungal pathogens is linked to environmental and developmental regulation, *A. fumigatus* gliotoxin production being regulated by BrlA, AbaA and WetA and *R. delemar* mucoricin being synthesized under aerobic rather than anaerobic conditions.^132^, ^133^, ^134^

## 4 COMMON FUNGAL FITNESS ATTRIBUTES THAT PROTECT AGAINST ENVIRONMENTAL CHALLENGES

Virulence factors are defined as fungal components that directly influence the host,^135^ but fungal pathogenicity also depends upon the ‘Fitness Attributes’ that promote the physiological robustness of the fungal cell within host niches.^90^ Fitness attributes include adaptive responses to fundamental environmental challenges that have been faced by microbes for billions of years. The challenges include, for example, changes in the availability of nutrients and essential micronutrients and fluctuations in ambient temperature, water balance, pH and oxygen levels. Consequently, evolutionarily divergent microbes share evolutionarily conserved responses to these ancient environmental challenges. They display the ability to switch between different carbon and nitrogen sources, to scavenge essential micronutrients such as iron and zinc from the surroundings, to assimilate osmolytes to restore turgor pressure, to synthesize antioxidants and detoxify reactive oxygen species and to repair damage to proteins and DNA. Furthermore, core regulators of these responses tend to be conserved across the fungal kingdom and some even in human cells. Examples include the Hog1 stress activated protein kinases (SAPKs) that activate osmotic and other stress responses,^2^, ^7^, ^136^ the AP‐1‐like transcription factors and Skn7 response regulators that drive oxidative stress responses,^137^, ^138^, ^139^, ^140^, ^141^, ^142^ the Slt2/Mkc1 mitogen activated protein kinases (MAPKs) critical for cell wall remodelling,^143^, ^144^, ^145^ the Zrt1/2 transporters involved in zinc scavenging,^146^ the Snf1 AMP kinases and Mig1/CreA transcription factors that control carbon assimilation,^147^, ^148^, ^149^ the Gcn4/CpcA transcription factors that regulate amino acid biosynthesis,^150^, ^151^, ^152^ and the PKA orthologues that control growth, morphogenesis and stress adaptation.^5^, ^153^, ^154^, ^155^, ^156^

Model fungi and fungal pathogens display differences in their adaptive responses. For example, they differ with respect to the sensitivities and amplitudes of their metabolic adaptation and stress responses. During their evolution there has also been regulatory rewiring which has led to the functional reassignment of certain regulators. Presumably these changes reflect the selective pressures that these fungal species have encountered in their respective niches. For example, the Hog1 SAPK, which activates the osmotic stress response in *S. cerevisiae*, has been co‐opted to regulate responses to oxidative stress, heavy metal stress, quorum sensing molecules and virulence in *C. albicans*.^3^, ^7^, ^157^ The Gal4 transcription factor, which controls galactose assimilation in *S. cerevisiae*, regulates central carbon metabolism in *C. albicans*.^158^ The Msn2/4 transcription factors activate the core transcriptional response to stress in *S. cerevisiae* but control the weak acid stress response in *C. albicans*.^159^, ^160^, ^161^, ^162^, ^163^ Also, when *S. cerevisiae* cells are exposed to glucose, catabolite inactivation drives the ubiquitin‐mediated degradation of enzymes required for gluconeogenesis and the assimilation of alternative, less favourable carbon sources.^164^, ^165^ In contrast, catabolite inactivation appears to have been relaxed in *C. albicans*, allowing these enzymes to persist when glucose is available.^166^ This means that while *S. cerevisiae* turns to alternative carbon sources only once available sugars have been depleted, *C. albicans* can simultaneously utilize sugars and alternative carbon sources such as fatty acids or lactate,^166^, ^167^ and this contributes to the virulence of this fungus.^167^ Therefore, while evolutionarily conserved core signalling modules regulate stress and nutrient adaptation, these modules and their outputs have been tuned in response to niche‐specific evolutionary pressures.

It has been attractive to dissect specific signalling pathways in isolation with a view to defining their roles in controlling specific stress and metabolic responses. This can lead to the misleading impression that these signalling pathways act in isolation. However, there is abundant evidence to indicate that stress and metabolic signalling pathways are intimately linked.^168^ For example, the core stress response in *S. cerevisiae* is downregulated in response to glucose via PKA signalling.^154^, ^169^ Key stress regulators in *C. albicans* also modulate metabolic genes (e.g. Hog1, Cap1, Hsf1) and core metabolic regulators also regulate stress genes (e.g. Gcn4, Mig1, Rbf1).^142^, ^149^, ^170^, ^171^, ^172^, ^173^, ^174^, ^175^ Furthermore, second messengers such as cyclic AMP and cyclic GMP are integral components of the metabolic network. Therefore, there is considerable crosstalk between the individual signalling pathways that control stress and metabolic adaptation. This means that, in reality, these pathways are intimately linked within a complex Resilience Network of signalling pathways (Figure 1). The core regulators described above and many others besides, represent hubs in this Resilience Network.

**FIGURE 2:**
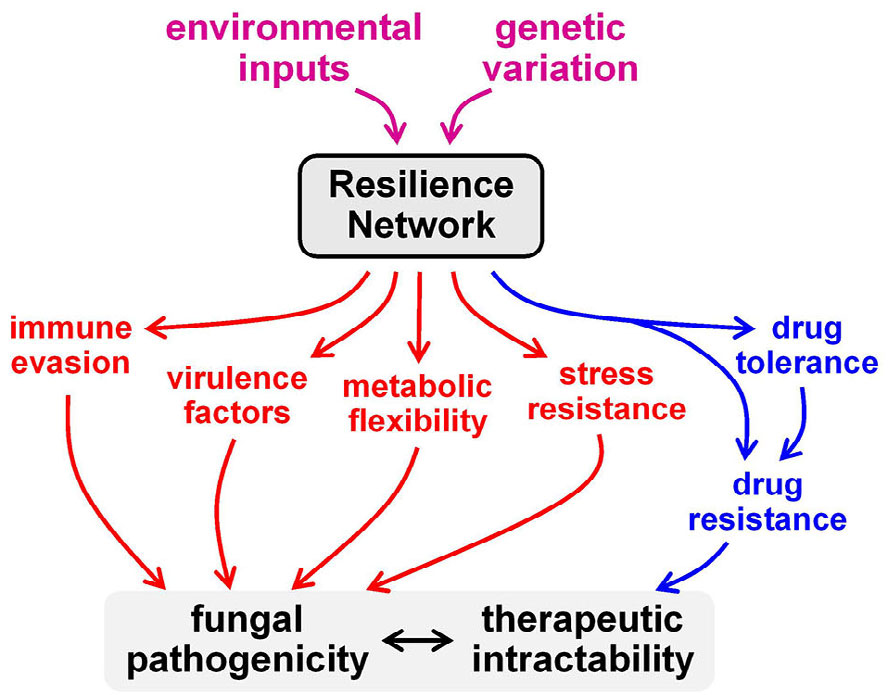
The Resilience Network influences the pathogenicity and therapeutic tractability of fungal pathogens. The Resilience Network responds to environmental inputs and its functionality is influenced by genetic variation (Figure 1). In addition to controlling stress resistance and metabolic flexibility, the Resilience Network regulates the expression of fungal virulence factors and the activation of fungal immune evasion strategies. These properties strongly influence fungal pathogenicity. In addition, the Resilience Network strongly influences the tolerance of fungal cells to antifungal drugs and drug tolerance permits the evolution of drug resistance.^8^, ^14^ Genetic variation between fungal species and between clinical isolates of these species influences the functionality of the Resilience Network and, consequently, their tolerance of and resistance to environmental stresses and antifungal drugs. Furthermore, the functionality of the Resilience Network is probably influenced by stochastic differences in the expression of key regulators and hence is likely to underlie the ability of subsets of fungal cells within clonal populations to evade antifungal immunity or tolerate antifungal drugs (see text)

Numerous lines of evidence indicate that this concept extends to antifungal drug resistance and tolerance. For example, the genome sequencing of drug resistant clinical isolates that have evolved in patients undergoing protracted antifungal therapy has revealed that the evolved resistance is associated with mutations in nutrient assimilation and stress pathway genes as well as with mutations that affect drug targets and efflux pumps.^176^ The imposition of stress has been shown to accelerate the rate at which *C. albicans* cells evolve resistance to antifungal drugs.^177^ The stress induced molecular chaperone, Hsp90, promotes the emergence of drug resistance in evolutionarily diverse fungi,^8^ and drug resistance is synergistically compromised when the cell wall integrity pathway, calcineurin signalling or Hsp90 functionality is perturbed.^9^, ^12^ Furthermore, cellular adaptation to changes in nutrients or stress imposition strongly influences the resistance of *Candida* to antifungal drugs.^8^, ^118^ Significantly, this is thought to be driven by the impact of stress pathways on drug tolerance.^178^ Therefore, the fungal Resilience Network impacts drug tolerance and resistance as well as stress and nutrient adaptation (Figure 2).

To summarize, it is apparent that, despite the evolutionary diversity of fungal pathogens, they tend to display broadly similar fitness attributes that empower the physiological robustness of fungal pathogens during infection. Furthermore, the regulation of these virulence factors and fitness attributes and the therapeutic tractability of fungal strains, are intertwined within a Resilience Network that is based on the crosstalk between individual signalling modules. Because cell growth and division, metabolism and stress responses are fundamental processes that are evolutionarily conserved in most organisms, many features of the Resilience Network that links these processes are likely to be conserved across fungal pathogens of humans, despite their evolutionary diversity. In other words, although the relative strength of specific pathway connections will vary, the general architecture of Resilience Networks across divergent fungal pathogens is likely to display a reasonable level of conservation.

## 5 THE ARCHITECTURE OF THE RESILIENCE NETWORK

Based on the above arguments, a major challenge for the future is to define the architecture of the Resilience Network. But what is the Resilience Network? So far, I have largely inferred that this network represents the system of interconnected signalling pathways within a fungal pathogen. However, to underlie cellular fitness, this network must include structural proteins, repair and detoxification enzymes, cell wall enzymes and polymers, molecular machines such as the ribosome and respiratory chain, energy in the form of ATP, antioxidants such as glutathione and multifarious other molecules in addition to the signalling molecules that regulate their levels and activities. Therefore, the Resilience Network must ultimately incorporate information about the proteome and metabolome as well as the transcriptome (Figure 1). It must include data about the activities of key regulators and must include key molecular links between the individual metabolic, regulatory and structural modules that make up the network.

These individual modules, their interconnections and their relative contributions to the fitness of the fungal cell need to be defined. This type of information would help to elaborate the structure of the Resilience Network and its functionality under a given set of circumstances. Historically, the adaptive responses of fungal pathogens have generally been dissected under ‘standard’ laboratory conditions that bear little resemblance to microenvironments in host niches.^7^, ^179^, ^180^, ^181^, ^182^ Therefore, as a starting point, the structure of the Resilience Network should be elaborated under defined in vitro conditions that relate to host niches, possibly in Roswell Park Memorial Institute (RPMI) medium, for example. The next step might be to compare this structure with those present in host niches. For *C. albicans* this might involve analysing the Resilience Network in cells colonizing the oral and vaginal epithelia, the colon and the bloodstream, whereas fungal cells from the lung and brain would be more appropriate for *C. neoformans*. These experiments would provide important insights into how the Resilience Network changes its architecture in fungal populations occupying these different host niches.

Great opportunities will arise from an understanding of the architecture of the fungal Resilience Network, how it adapts to different host niches and how this impacts fungal virulence and resilience towards innate immune defences and antifungal drugs. In particular, this will highlight nodes in the network that are critical for fungal resilience within host niches. These nodes, be they specific regulatory hubs, molecular machines or metabolic enzymes, may represent novel targets for therapeutic intervention (Figure 2). Some of these modules will interact with those that are targeted by antifungal drugs in clinical use, such as polyenes and azoles (ergosterol biosynthesis and membrane integrity) and echinocandins (β‐(1,3)‐glucan synthesis and cell wall functionality).^12^, ^183^, ^184^ Inhibitors of these Resilience Network modules will have the potential to act synergistically with antifungal to inhibit the growth or kill fungal pathogens more effectively. There are clear precedents for this. For example, pharmacological inhibitors of Hsp90 or PKC signalling act synergistically with fluconazole to block the growth of *C. albicans* and, significantly, imparts this fungistatic drug with fungicidal activity.^9^ Of course, many strict criteria must be fulfilled before a molecule can be used therapeutically. Nevertheless, it is work noting that some inhibitors of Hsp90 and PKC signalling are already in therapeutic use.^12^

The holistic views of fungal adaptation mechanisms provided by the Resilience Network are likely to reveal additional examples of potentially useful approaches towards combination therapies.^12^ This is important because combination therapies are proving effective in the treatment of complex conditions, can retard the emergence of drug resistance and may help to control drug resistant isolates.^185^, ^186^, ^187^, ^188^ In addition, the comparison of Resilience Networks between evolutionarily divergent fungi is likely to reveal common points of fragility that represent potential targets for broad spectrum antifungal drugs.

## 6 THE DYNAMISM OF THE RESILIENCE NETWORK

As discussed above, the microenvironments occupied by fungal pathogens are in a constant state of flux as niches are altered by the growth of fungal cells and the influx of immune infiltrates. Consequently, the Resilience Network itself must be in a constant state of flux as it detects and responds to these changes in the microenvironment. Dynamic changes in the Resilience Network are likely to provide mechanisms of fungal escape from pharmacological or genetic intervention, in the same way as elevated chitin synthesis provides a means of escaping the inhibitory effects of echinocandins.^189^, ^190^, ^191^ These dynamics are also likely to provide escape from immune attack, passively through the modulation of cellular resistance to antifungal killing mechanisms and actively through pre‐emptive defensive responses against these mechanisms.^11^, ^13^, ^35^, ^85^ Therefore, having established the network architecture, the next challenge will be to define the network dynamics. Time‐series measurements of the activities of key regulators and the levels of critical metabolites would lie at the heart of this challenge.

Additional routes of escape from pharmacological insults to the Resilience Network exist for fungal pathogens. Clinical isolates of *C. albicans* display a high degree of genetic and phenotypic variability that includes differences in drug resistance and tolerance and the extent to which isolates are recognized by innate immune cells.^11^, ^192^, ^193^ Also, when placed under selective pressure, fungal pathogens assimilate mutations that enhance their fitness under these conditions.^176^, ^194^, ^195^ Defining mutations in clinical isolates or micro‐evolved strains that influence the functionality of the Resilience Network and that underlie their resistance, their tolerance phenotypes and their immune visibility, will enhance our understanding of these processes and, significantly the strengths and fragilities of the Resilience Network that underlies them.

In addition, individual cells within clonal populations of *C. albicans* display considerable heterogeneity with respect to their immune visibility and their tolerance to environmental stresses and antifungal drugs.^14^, ^196^, ^197^ Stochastic differences between individual cells that affect the status of the Resilience Network, such as variations in the levels or activities of key regulators, are bound to contribute to this population heterogeneity. Therefore, determining which stochastic variations exert the greatest impact upon the Resilience Network will advance our awareness of the nodes that contribute most to its elasticity or that offer points of fragility.

## 7 TECHNOLOGICAL INNOVATIONS LIKELY TO EMPOWER A DEEP UNDERSTANDING OF RESILIENCE NETWORK ARCHITECTURE

Major advances in biology have often been dependent upon significant technological innovations. Paul Nurse describes how Robert Hooke's refinement of the compound microscope led to the discovery of the cell.^1^ Fred Sanger's and Walter Gilbert's development of DNA sequencing technologies^198^, ^199^ opened the door the elaboration of gene structure and ultimately to the sequences of whole genomes.^200^, ^201^, ^202^ The subsequent development of next generation sequencing^203^, ^204^ has led to dramatic advances in our understanding of fungal evolution, epidemiology and the emergence of drug resistance.^176^, ^192^, ^193^, ^205^ Clearly, future technical advances will accelerate the elaboration of Resilience Network architecture and enhance our understanding of how this network influences fungal resistance to antifungal immunity and therapeutic intervention.

Fungal pathogens are generally less experimentally tractable than model yeasts such as *S. cerevisiae* and *S. pombe*. Focusing on *C. albicans*, for many decades this major fungal pathogen was considered asexual, which severely limited the genetic dissection of its pathobiology.^206^, ^207^ This fungus was also found to display non‐standard codon usage, which constrained the exploitation of reporter‐based screens.^208^, ^209^ To a certain extent these challenges were circumvented by the development of codon‐optimized reporters and accurate tools for gene manipulation.^210^, ^211^, ^212^, ^213^, ^214^, ^215^, ^216^, ^217^, ^218^ Significantly, the emergence of genome sequence data^219^ empowered the development of transcript profiling and other genome‐wide tools for *C. albicans*.^170^, ^171^, ^220^, ^221^, ^222^, ^223^, ^224^, ^225^ Also, the discovery of efficient mating and concerted chromosome loss permitted the completion of a parasexual cycle for *C. albicans*.^207^, ^226^, ^227^, ^228^, ^229^ The revelation of haploid forms of *C. albicans* and subsequent generation of transposon libraries also represented major steps forward.^230^, ^231^, ^232^ Yet we still lack a complete collection of barcoded gene knockouts for *C. albicans* or for any fungal pathogen as far as I am aware. This kind of resource has proven invaluable for the *S. cerevisiae* research community permitting, for example, efficient comparisons of relative fitness for large pools of mutants in competition assays as well as global transcriptomic, proteomic and metabolomic analyses of these mutants.^233^, ^234^ Clearly, the availability of a complete collection of null mutants for *C. albicans* would dramatically accelerate the elaboration of the Resilience Network in this pathogen.

Looking further ahead, the lack of a complete sexual cycle remains a major obstacle for the *C. albicans* research community. The ability to combine genetics with genomics in *S. cerevisiae* has proven extremely powerful. In particular, this facilitated the description of the genetic landscape of the yeast cell through the comprehensive dissection of synthetic genetic interactions.^235^, ^236^ Significantly, genetics has extended the reach of genomics beyond the analysis of gene deletions to include single nucleotide polymorphisms (SNPs) and small insertions and deletions (indels). This is important because while essential genes can be represented in mutant libraries by loss‐ and gain‐of‐function mutations caused by SNPs and indels, they are not included in the collections of gene deletion mutants that are generally exploited for genome‐wide loss‐of function screens. Yet essential genes tend to be more highly conserved than non‐essential genes, they tend to be more highly connected within networks and they represent attractive antifungal targets.^237^, ^238^, ^239^, ^240^ Therefore, approaches such as bulk segregant analysis (BSA), which screens for sequence polymorphisms that are associated with a specific phenotype, are likely to prove a rich source of information about the genes—essential or non‐essential—that contribute to fungal pathogenicity and drug resistance. Briefly, BSA involves sequential cycles of genetic crossing and the selection, at each stage of the process, for progeny with the phenotype of interest, whether it be drug or stress resistance, for example. After multiple rounds of crossing, the pooled genome sequence for the resistant segregants is compared with that for the pool of sensitive segregants with a view to identifying mutations that are significantly enriched in the resistant pool.^241^ Importantly, this powerful approach has highlighted new genes that contribute to *S. cerevisiae* phenotypes that had already been studied in great detail.^242^, ^243^, ^244^, ^245^ Furthermore, BSA is capable of highlighting quantitative trait loci at single nucleotide resolution.^241^

**FIGURE 3:**
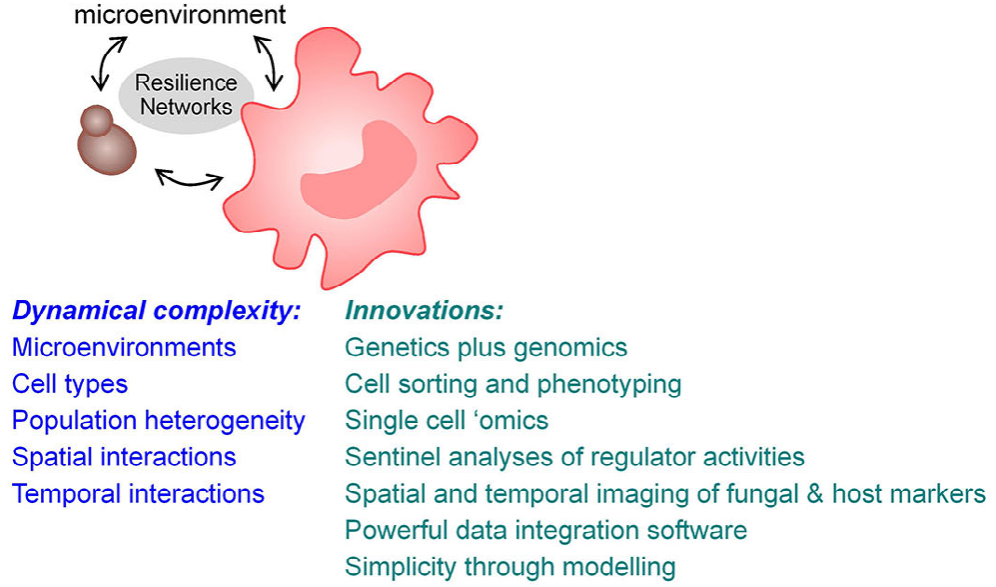
Challenges and potential ways forward. Host–fungus interactions are both complex and dynamic and, although the focus of this perspective is on the fungus, the Resilience Networks of both the fungus and the host lie at the heart of these interactions. The dynamic complexity of these interactions is influenced by the local microenvironment, the fungus, the host cell types, the degree of population heterogeneity of these cell types and the spatial organization and temporal dynamics of these interactions. Potential advances that will help to disentangle this enormous complexity are likely to include the engineering of sexual cycles in fungal pathogens to empower the application of fungal genetics alongside fungal genomics. In addition, cytometric cell sorting based on advanced markers of cell type and activity will permit accurate phenotyping of relevant cell types and populations. Single cell proteomics and possibly single cell metabolomics will complement single cell genomics and transcriptomics. Deep proteomic sentinel analyses will permit parallel quantification of the activities of Resilience Network modules within cells. Advanced immunohistochemical imaging will permit spatial and temporal relationships between specific host and fungal cell types within tissues to be elaborated. Powerful new software capable of integrating multidimensional ‘omic’ datasets will be required to achieve all of this and elegant modelling will be essential to reveal the simplicity that underlies this complexity (see text)

Clearly, the application of BSA to *C. albicans* pathobiology is hindered by the lack of a complete sexual cycle in this species. However, the ability to engineer a sexual cycle in *C. glabrata* ^246^ suggests that this might also be possible in *C. albicans*. An engineered sexual cycle, combined with approaches to enhance genetic recombination in *C. albicans*,^247^, ^248^ would open the door to BSA and other powerful genetics‐based approaches in this pathogen (Figure 3). These, together with new ultra‐fast proteomic^249^, ^250^ and, hopefully, metabolomic analyses of defined mutants would provide deep datasets that would inform the architecture of the Resilience Network. This would require, however, machine learning and advanced software capable of integrating genes, transcripts and proteins with the molecular machines they contribute to and/or the reactions they catalyse as well as the corresponding metabolites.^251^ No doubt, in the future, the application of the accurate protein folding algorithms of DeepMind^252^ will also be exploited to strengthen interactions within the Resilience Network and to provide evolutionary comparisons with analogous networks in other fungal pathogens. In this way, common points of fragility likely to affect fungal virulence, PAMP synthesis and exposure, stress resistance or drug tolerance may be identified.

## 8 INNOVATIONS TO DEFINE THE DYNAMICS AND SPATIAL ACTIVATION OF THE NETWORK

The above innovations will permit the elaboration of Resilience Network architecture—the inputs, the regulators, the components and their interactions. The next challenges will be to define the dynamics of the Resilience Network, its spatial activation and its impact upon population heterogeneity in the context of host niches and the interactions between fungal cells and specific host cell types (Figure 3).

The definition of network dynamics will be dependent upon time‐series measurements of key regulators and enzymes, their activities and the levels of critical metabolites and for this, advanced, targeted proteomics and metabolomics will be required. The way forward may be offered by innovations that permit the multiplexing of high throughput sentinel assays that quantify the relative levels of post‐translationally modified, activated proteins.^253^, ^254^, ^255^, ^256^ Similarly, innovations that extend current metabolomic capabilities to follow the concentrations of multiple classes of metabolite in real time would be a major step forward.^257^, ^258^

Cytometry provides a classical approach to the analysis of population heterogeneity. Therefore, the development of a library of differentially labelled antibody fragments that are specific for the post‐translationally modified, active and native versions of key regulators would permit rapid analysis of the Resilience Network in individual cells within clonal fungal populations. By applying fluorescence activated cell sorting, the behaviours of individual cells could be examined in detail. The transcriptomes of individual cells are already accessible through single cell RNA sequencing,^259^, ^260^ and single cell proteomics is now feasible.^261^ Therefore, the answers to questions such as these are within reach. How do different Network modules respond in individual cells to specific immunological, pharmacological or genetic insults? Are compensatory behaviours dependent on specific regulatory hubs? Can these hubs be targeted chemically to trigger synergistic killing of fungal cells?

The spatial definition of fungal Resilience Network behaviours in the context of host niches and local immune infiltrates is a major challenge for the future. Yet recent technological innovations appear to bring this exciting goal within reach (Figure 3). The MACSima imaging platform is reported to permit the analysis of hundreds of markers on single tissue sections.^262^ Using this system, markers for specific immune cell types could be combined with markers for the activation of specific modules in the fungal Resilience Network. This approach would provide invaluable spatial information, within tissue samples, about the nature of immune responses to fungal colonization and how the fungal cells respond locally to immune attack. By including specific bacterial markers, this principle could be extended to examine the influence of local microbiotas upon host–fungus interactions. This information would move our understanding of host–fungus–microbiota interactions to a completely new dimension.^15^

## 9 CONQUERING COMPLEXITY WITH SIMPLICITY

As Bernhard Hube has suggested, host–pathogen interactions are complicated! Indeed, in focussing on the fungal pathogen, this article has not addressed other important aspects of this complexity such as the diversity of immune cell types, their antifungal weaponry and their interactions.^15^, ^76^, ^263^, ^264^ Nevertheless, understanding the fungal Resilience Network will provide critical insights into how pathogenic fungi evade immunity, how they are adept at colonizing specific niches, why some fungal isolates are tolerant to antifungal drugs and why some fungal pathogens persist within specific niches. The fungal Resilience Network will also present valuable opportunities to develop new antifungal targets. Based on strong precedents,^9^, ^12^, ^184^ inhibitors of some of these targets are likely to synergise with current antifungal drugs and hence empower the development of combination therapies strengthen our limited armoury of antifungal drugs^265^ and possibly retard the emergence of antifungal drug resistance.^188^

## FUNDING INFORMATION

This work was funded by a programme grant from the UK Medical Research Council (www.mrc.ac.uk: MR/M026663/2) and by the Medical Research Council Centre for Medical Mycology at the University of Exeter (MR/N006364/2). The funder had no role in this study, the decision to publish or the preparation of the manuscript.

## CONFLICT OF INTEREST

The author has declared no conflicts of interest for this article.

## Data Availability

This review contains no new data.
